# Supplementary material for: Association between physical multimorbidity and common mental health disorders in rural and urban Malawian settings: Preliminary findings from Healthy Lives Malawi long-term conditions survey
Source: PLOS Glob Public Health. 2024 Apr 4;4(4):e0002955. doi: 10.1371/journal.pgph.0002955 (PMC10994288; doi:10.1371/journal.pgph.0002955)
Supplement: S1 Appendix — (DOCX) [file pgph.0002955.s001.docx]

| **S1 Appendix Association between sociodemographic and lifestyle factors and depression and anxiety scores** | | | |
| --- | --- | --- | --- |
| Variable | | PHQ-9, mean (SD) | GAD7, mean (SD) |
| Site | |  |  |
|  | Karonga (rural) | 1.6 (2.8) | 1.3 (2.5) |
|  | Lilongwe (Urban) | 2.8 (3.5) | 1.9 (2.9) |
|  | t, *p*-value | - 15.5, <0.001 | -8.8. <0.001 |
| Age group (years) ^a^ | |  |  |
|  | 15 – 17 | 1.1 (2.0) | 0.8 (1.7) |
|  | 18 – 29 | 2.0 (3.1) | 1.5 (2.6) |
|  | 30 – 39 | 2.1 (3.3) | 1.6 (2.9) |
|  | 40 – 49 | 1.8 (3.0) | 1.4 (2.5) |
|  | 50 – 59 | 1.7 (2.9) | 1.4 (2.5) |
|  | ≥60 | 1.5 (2.8) | 1.1 (2.5) |
|  | F, *p*-value | 21.1, <0.001 | 18.1, <0.001 |
| Sex | |  |  |
|  | Male | 1.7 (2.9) | 1.3 (2.4) |
|  | Female | 1.9 (3.0) | 1.5 (2.7) |
|  | t, *p*-value | - 2.2 , 0.031 | -4.4. <0.001 |
| Education attainment ^a^ | |  |  |
|  | Not completed any level | 1.7 (2.8) | 1.4 (2.5) |
|  | Primary | 1.8 (3.1) | 1.3 (2.7) |
|  | Junior secondary | 1.9 (2.8) | 1.4 (2.5) |
|  | Senior secondary & post-secondary | 2.2 (3.2) | 1.6 (2.6) |
|  | Missing | 1.6 (2.5) | 1.1 (2.1) |
|  | F, *p*-value | 10.0, <0.001 | 2.9, 0.023 |
| Marital status ^a^ | |  |  |
|  | Never married | 1.6 (2.7) | 1.2 (2.2) |
|  | Married | 1.9 (3.1) | 1.5 (2.7) |
|  | Divorced/separated/Widowed | 2.0 (3.1) | 1.6 (2.8) |
|  | F, *p*-value | 9.0, <0.001 | 14.3, <0.001 |
| Employment ^a^ | |  |  |
|  | Not employed | 2.1 (3.2) | 1.5 (2.7) |
|  | Full-time student | 1.4 (2.4) | 1.0 (2.0) |
|  | Self/irregular | 1.8 (2.9) | 1.4 (2.6) |
|  | Employed/regular | 2.2 (3.1) | 1.5 (2.6) |
|  | F, *p*-value | 19.4, <0.001 | 13.9, <0.001 |
| Smoking status | |  |  |
|  | Never | 1.8 (2.9) | 1.4 (2.6) |
|  | Former/Current | 2.5 (3.3) | 1.6 (2.9) |
|  | t, *p*-value | -3.5, <0.001 | -2.5, 0.013 |
| Alcohol consumption | |  |  |
|  | Not in last year | 1.8 (2.9) | 1.3 (2.5) |
|  | In last year | 2.1 (3.1) | 1.6 (2.7) |
|  | Missing | 5.8 (5.5) | 1.7 (2.1) |
|  | F, *p*-value | 16.7, <0.001 | 9.2, <0.001 |
| Physical activity ^a^ | |  |  |
|  | Not met strength guidelines | 1.8 (3.0) | 1.4 (2.6) |
|  | Met strength guidelines | 1.9 (2.9) | 1.4 (2.4) |
|  | Missing | 0.8 (1.6) | 0.5 (1.3) |
|  | F, *p*-value | 9.9, <0.001 | 8.4, <0.001 |
| Body mass index (kg/m²) | |  |  |
|  | Underweight | 1.6 (2.8) | 1.2 (2.5) |
|  | Normal | 1.8 (3.0) | 1.4 (2.6) |
|  | Overweight | 1.9 (2.9) | 1.4 (2.6) |
|  | Obese | 2.0 (2.9) | 1.5 (2.6) |
|  | Missing | 2.0 (3.5) | 1.2 (2.5) |
|  | F, *p*-value | 2.3 , 0.059 | 1.9, 0.107 |
| Number of physical health conditions | |  |  |
|  | None | 1.5 (2.6) | 1.2 (2.2) |
|  | One | 2.2 (3.2) | 1.7 (3.0) |
|  | Two | 2.6 (3.7) | 2.0 (3.3) |
|  | Three or more | 3.1 (3.9) | 2.2 (3.4) |
|  | Incomplete data | 1.7 (2.7) | 1.3 (2.5) |
|  | F, *p*-value | 50.2 , <0.001 | 41.8, <0.001 |
| F means F-value from ANOVA  t means t-value from t-test  ^a^ Bonferroni post-hoc test was done for significant ANOVA | | | |
