# Supplementary material for: Association between physical multimorbidity and common mental health disorders in rural and urban Malawian settings: Preliminary findings from Healthy Lives Malawi long-term conditions survey
Source: PLOS Glob Public Health. 2024 Apr 4;4(4):e0002955. doi: 10.1371/journal.pgph.0002955 (PMC10994288; doi:10.1371/journal.pgph.0002955)
Supplement: S2 Appendix — (DOCX) [file pgph.0002955.s002.docx]

| **S2 Appendix Common combinations of multiple health conditions** | |
| --- | --- |
| **Health condition combination** ^a^ | **n (%)** |
| *Two conditions (n = 912)* |  |
| Hypertension + disability | 164 (18%) |
| Hypertension + chronic pain | 127 (14%) |
| Disability + chronic pain | 87 (10%) |
| Hypertension + HIV | 78 (9%) |
| Hypertension + asthma | 66 (7%) |
| Hypertension + diabetes | 60 (6%) |
| *Three conditions (n = 263)* |  |
| Hypertension + disability + chronic pain | 64 (24%) |
| Hypertension + disability + HIV | 22 (8%) |
| Diabetes + hypertension + disability | 21 (8%) |
| Hypertension + disability + asthma | 14 (5%) |
| Hypertension+ disability + stroke | 13 (5%) |
| Disability + asthma + chronic pain | 13 (5%) |
| *Four conditions (n = 47)* |  |
| Diabetes + hypertension + disability + chronic pain | 11 (23%) |
| Hypertension + disability + asthma + chronic pain | 6 (13%) |
| Hypertension + disability + heart disease + chronic pain | 3 (6%) |
| *Five conditions (n = 10)* |  |
| Diabetes, hypertension, disability, heart disease, chronic pain | 2 (20%) |
| Diabetes, hypertension, disability, asthma, chronic pain | 2 (20%) |
| ^a^  Only combinations of conditions with prevalence ≥ 5% are presented in table S2 | |
