# Supplementary material for: Association between physical multimorbidity and common mental health disorders in rural and urban Malawian settings: Preliminary findings from Healthy Lives Malawi long-term conditions survey
Source: PLOS Glob Public Health. 2024 Apr 4;4(4):e0002955. doi: 10.1371/journal.pgph.0002955 (PMC10994288; doi:10.1371/journal.pgph.0002955)
Supplement: S3 Appendix — (DOCX) [file pgph.0002955.s003.docx]

| **S3 Appendix Sensitivity analysis of the association between number of physical health conditions and depression and anxiety scores, stratified by age** | | | | | | | | | | | |
| --- | --- | --- | --- | --- | --- | --- | --- | --- | --- | --- | --- |
|  |  | Depression score ^a^ | | | | | | | | | |
|  |  | 15 – 17 years | | 18 – 29 years | | 30 – 39 years | | 40 – 49 years | | ≥50 years | |
| Number of physical conditions | | B coefficient (95% CI) | P-value | B coefficient (95% CI) | P-value | B coefficient (95% CI) | P-value | B coefficient (95% CI) | P-value | B coefficient (95% CI) | P-value |
|  | None | Ref. |  | Ref. |  | Ref. |  | Ref. |  | Ref. |  |
|  | One | 0.65 (0.07 – 1.23) | 0.028 | 1.02 (0.74 – 1.29) | <0.001 | 0.91 (0.56 – 1.26) | <0.001 | 0.30 (-0.07 – 0.69) | 0.113 | 0.45 (0.07 – 0.82) | 0.019 |
|  | Two | 1.28 (0.34 – 2.22) | 0.008 | 1.51 (1.02 – 2.01) | <0.001 | 1.57 (1.03 – 2.11) | <0.001 | 1.57 (1.09 – 2.05) | <0.001 | 0.56 (0.16 – 0.96) | 0.007 |
|  | Three or more | - | - | 3.80 (2.46 – 5.13) | <0.001 | 2.56 (1.29 – 3.82) | <0.001 | 1.96 (1.20 – 2.71) | <0.001 | 1.52 (1.01 – 2.03) | <0.001 |
|  |  | Anxiety score ^b^ | | | | | | | | | |
| Number of physical conditions | |  |  |  |  |  |  |  |  |  |  |
|  | None | Ref. |  | Ref. |  | Ref. |  | Ref. |  | Ref. |  |
|  | One | 0.82 (0.32 – 1.32) | 0.001 | 0.74 (0.49 – 0.97) | <0.001 | 0.84 (0.52 – 1.67) | <0.001 | 0.39 (0.08 – 0.70) | 0.013 | 0.30 (-0.04 – 0.64) | 0.079 |
|  | Two | 1.57 (0.76 – 2.38) | <0.001 | 1.59 (1.15 – 2.01) | <0.001 | 1.18 (0.68 – 1.67) | <0.001 | 1.18 (0.78 – 1.58) | <0.001 | 0.51 (0.15 – 0.87) | 0.006 |
|  | Three or more | - | - | 3.15 (1.98 – 4.31) | <0.001 | 0.65 (-0.50 – 1.81) | 0.267 | 1.78 (1.15 – 2.41) | <0.001 | 0.97 (0.52 – 1.43) | <0.001 |
|  |  |  |  |  |  |  |  |  |  |  |  |
| ^a^ Patient Health Questionnaire-9 depression score , measured on continuous scale from 0 – 27.  ^b^ General Anxiety Disorder-7 anxiety score, measured on a continuous scale from 0 – 21.  Model I: Adjust for sex, age, site education, and employment status, marital status.  Model II: Adjust for the variables in Model I plus physical activity, BMI, alcohol status, and smoking status | | | | | | | | | | | |
