# Supplementary material for: Association between physical multimorbidity and common mental health disorders in rural and urban Malawian settings: Preliminary findings from Healthy Lives Malawi long-term conditions survey
Source: PLOS Glob Public Health. 2024 Apr 4;4(4):e0002955. doi: 10.1371/journal.pgph.0002955 (PMC10994288; doi:10.1371/journal.pgph.0002955)
Supplement: S4 Appendix — (DOCX) [file pgph.0002955.s004.docx]

| **S4 Appendix Distribution of sociodemographic characteristics by availability of multimorbidity data** | | | | |
| --- | --- | --- | --- | --- |
| Variable | | Sufficient^+^ data (n=8754) | Incomplete multimorbidity data (n=1094) | *p*-value ^a^ |
|  |  | n (%) | n (%) |  |
| Sex | |  |  | <0.001 |
|  | Male | 3722 (43%) | 565 (52%) |  |
|  | Female | 5032 (57%) | 530 (48%) |  |
| Age group (years) | |  |  | <0.001 |
|  | 15 – 17 | 857 (10%) | 285 (26%) |  |
|  | 18 – 29 | 3332 (38%) | 411 (38%) |  |
|  | 30 – 39 | 1782 (20%) | 124 (11%) |  |
|  | 40 – 49 | 1293 (15%) | 90 (8%) |  |
|  | 50 – 59 | 725 (8%) | 57 (5%) |  |
|  | ≥60 | 765 (9%) | 128 (12%) |  |
| Site | |  |  | <0.001 |
|  | Lilongwe | 1602 (18%) | 317 (29%) |  |
|  | Karonga | 7152 (82%) | 778 (71%) |  |
| Education attainment | |  |  | <0.001 |
|  | Not completed any level | 4394 (50%) | 630 (58%) |  |
|  | Primary | 1441 (17%) | 163 (15%) |  |
|  | Junior secondary | 1232 (14%) | 110 (10%) |  |
|  | Senior secondary | 1057 (12%) | 118 (11%) |  |
|  | Post secondary | 491 (6%) | 48 (4%) |  |
|  | Missing | 139 (2%) | 26 (2%) |  |
| Employment status | |  |  | <0.001 |
|  | Not employed | 2385 (27%) | 311 (28%) |  |
|  | Full-time student | 1161 (13%) | 325 (30%) |  |
|  | Self or irregular | 4565 (52) | 398 (36%) |  |
|  | Employed or regular | 643 (7%) | 61 (6%) |  |
| Depressive symptoms score  Mean (SD) ^b^ | | 1.8 (3.0) | 1.7 (2.7) | 0.281 |
| Anxiety symptoms score  Mean (SD) ^b^ | | 1.4 (2.6) | 1.3 (2.5) | 0.467 |
| SD = standard deviation  ^a^ p-value from chi-square test unless specified.  ^b^ p-value from t-test  ^+^ Sufficient data if self-reported or measured with two or more conditions, irrespective of whether missing data on one or more conditions. | | | | |
