# Supplementary material for: Association between physical multimorbidity and common mental health disorders in rural and urban Malawian settings: Preliminary findings from Healthy Lives Malawi long-term conditions survey
Source: PLOS Glob Public Health. 2024 Apr 4;4(4):e0002955. doi: 10.1371/journal.pgph.0002955 (PMC10994288; doi:10.1371/journal.pgph.0002955)
Supplement: S5 Appendix — (DOCX) [file pgph.0002955.s005.docx]

| **S5 Appendix Sensitivity analysis of the association between number of physical health conditions and depression and anxiety scores, excluding those with missing data on any condition included in the multimorbidity definition** | | | | | | | |
| --- | --- | --- | --- | --- | --- | --- | --- |
|  |  |  | | Depression score ^a^ | |  | |
|  |  | Unadjusted Model | | Model I | | Model II | |
| Number of physical conditions | | B coefficient (95% CI) | P-value | B coefficient (95% CI) | P-value | B coefficient (95% CI) | P-value |
|  | None | Ref. |  | Ref. |  | Ref. |  |
|  | One | 0.57 (0.39 – 0.74) | <0.001 | 0.73 (0.55 – 0.90) | <0.001 | 0.74 (0.56 – 0.91) | <0.001 |
|  | Two | 1.13 (0.88 – 1.38) | <0.001 | 1.44 (1.18 – 1.71) | <0.001 | 1.47 (1.21 – 1.74) | <0.001 |
|  | Three or more | 1.53 (1.13 – 1.92) | <0.001 | 2.08 (1.67 – 2.50) | <0.001 | 2.19 (1.76 – 2.61) | <0.001 |
|  | *p* value for trend | <0.001 |  | <0.001 |  | <0.001 |  |
|  |  |  |  | Anxiety score ^b^ | |  |  |
| Number of physical conditions | |  |  |  |  |  |  |
|  | None | Ref. |  | Ref. |  | Ref. |  |
|  | One | 0.57 (0.43 – 0.70) | <0.001 | 0.61 (0.46 – 0.77) | <0.001 | 0.62 (0.46 – 0.77) | <0.001 |
|  | Two | 0.89 (0.71 – 1.07) | <0.001 | 1.14 (0.91 – 1.37) | <0.001 | 1.17 (0.93 – 1.40) | <0.001 |
|  | Three or more | 1.04 (0.74 – 1.33) | <0.001 | 1.58 (1.22 – 1.95) | <0.001 | 1.65 (1.28 – 2.03) | <0.001 |
|  | *p* value for trend | <0.001 |  | <0.001 |  | <0.001 |  |
| ^a^ Patient Health Questionnaire-9 depression score, measured on continuous scale from 0 – 27.  ^b^ General Anxiety Disorder-7 anxiety score, measured on a continuous scale from 0 – 21.  Model I: Adjust for sex, age, site education, and employment status, marital status.  Model II: Adjust for the variables in Model I plus physical activity, BMI, alcohol status, and smoking status | | | | | | | |
