# Supplementary material for: Association between physical multimorbidity and common mental health disorders in rural and urban Malawian settings: Preliminary findings from Healthy Lives Malawi long-term conditions survey
Source: PLOS Glob Public Health. 2024 Apr 4;4(4):e0002955. doi: 10.1371/journal.pgph.0002955 (PMC10994288; doi:10.1371/journal.pgph.0002955)
Supplement: Attachment — Submitted filename: Rebuttal Letter.docx [file pgph.0002955.s006.docx]

**Manuscript Number: PGPH-D-23-02092**

Dear Editor

**Rebuttal Letter**

We acknowledge the previous submission of this paper (previous Manuscript Number: PGPH-D-23-01510), which was intended to be included in the planned collection on multimorbidity in Africa. The journal decided to withdraw the manuscript because of its inability to identify a handling editor and suitable reviewers at that time.

Subsequently, we engaged in discussions with Julia Robinson, who recommended that we resubmit the paper. She expressed her willingness to assist in managing the review process and identifying appropriate reviewers. It is worth noting that Ms. Robinson is being considered as the potential handling editor for the collection.

In light of her guidance and support, we have re-submitted our paper in accordance with her advice. I am sure if this manuscript is queued to her she will handle it and help identify reviewers.

Kind Regards,

Owen Nkoka, PhD
