# Supplementary material for: Association between physical multimorbidity and common mental health disorders in rural and urban Malawian settings: Preliminary findings from Healthy Lives Malawi long-term conditions survey
Source: PLOS Glob Public Health. 2024 Apr 4;4(4):e0002955. doi: 10.1371/journal.pgph.0002955 (PMC10994288; doi:10.1371/journal.pgph.0002955)
Supplement: Attachment — Submitted filename: (PGPH-D-23-02092)_Response to reviewers_30012024.docx [file pgph.0002955.s007.docx]

**Ref.	PGPH-D-23-02092**

**Title: Association between physical multimorbidity and common mental health disorders in rural and urban Malawian settings: preliminary findings from Healthy Lives Malawi long-term conditions survey**

**Point-by-point responses**

**Journal Requirements:**

1. Please review your reference list to ensure that it is complete and correct. If you have cited papers that have been retracted, please include the rationale for doing so in the manuscript text or remove these references and replace them with relevant current references. Any changes to the reference list should be mentioned in the rebuttal letter that accompanies your revised manuscript. If you need to cite a retracted article, indicate the article’s retracted status in the References list and include a citation and full reference for the retraction notice.

***Response:***

We have reviewed the reference list, and we confirm that it is complete and correct. We have also added several references as per reviewer comments. The list of all added references has been highlighted in blue in the reference list.

**Reviewer #1: Reviewer Comments:**

Abstract
1. Lines 22-24:

Do the authors mean to add the word “conditions” at the end?
“Multimorbidity was defined as having two or more measured (diabetes, hypertension) or self-reported (diabetes, hypertension, disability, chronic pain, HIV, asthma, stroke, heart disease, and epilepsy).”

***Response:***

Thanks for the observation, we have revised the statement to include condition at the end. Now it reads as follows:

*Multimorbidity was defined as having two or more measured (diabetes, hypertension) or self-reported (diabetes, hypertension, disability, chronic pain, HIV, asthma, stroke, heart disease, and epilepsy conditions). (Page 2, line 23)*

2. Line 32:
“hypertension, and disability (18%) co-occurred most frequently,” the “18%” is better placed at the end of the word “frequently?”

***Response:***

We have revised and placed 18% after ‘frequently’. The statement now reads as follows:

*In adults with two conditions, hypertension, and disability co-occurred most frequently (18%), and… (Page 2, line 32)*

3. Line 33:
“hypertension, disability, and chronic pain (23%),” the “23%” is better placed at the end of the word “combination?”

***Response:***

We have revised as suggested and now it reads as follows:

*…and in those with three conditions, hypertension, disability, and chronic pain were the most common combination (23%). (Page 2, line 33).*

4. Lines 39-40:
Reads better: “and need to integrate mental and physical health service delivery in Malawi.

***Response:***

We have revised the statement to read:

…*our findings highlight the burden of multimorbidity and the need to integrate mental and physical health service delivery in Malawi… (Page 2, line 39)*

Introduction
1. Line 53:
Kindly add the reference after the multimorbidity definition. “Multimorbidity (MM), defined as the presence of two or more long-term conditions [ref],”

***Response:***

We have added a reference for the multimorbidity definition as suggested.

*Multimorbidity (MM), defined as the presence of two or more long-term conditions (LTCs) [8], (Page 3, line 53)*

2. Lines 55-56:
Is the mentioned 60% multimorbidity prevalence with reference to young adults or the general population within LMICs?
Please specify the prevalence in young adults (<25 years) and also older adults ((≥65 years).

***Response:***

We have revised this to make it more clear as follows:

*Multimorbidity, defined as the presence of two or more long-term conditions [8], is a concern for healthcare systems worldwide [9, 10]. In recent studies, the reported prevalence of multimorbidity in LMIC adults vary widely, with estimates ranging from 3.2% to 90.5% [11]. While the bulk of the multimorbidity burden is observed in those aged ≥60 years, emerging research shows multimorbidity even in younger adults (aged 18 – 49 years)[12-15], with a prevalence of 5.6% observed in Botswana in individuals aged 15 – 44 years [12]. The observed heterogeneity in multimorbidity prevalence estimates in different populations [13] and emerging burden in young adults highlights the need for, country-specific studies to determine high-risk and vulnerable groups and socioeconomic and lifestyle factors that influence burden [14] and early detection and intervention strategies to mitigate the long-term health impacts. (Page 3, line 52 – 61)*

3. Lines 63-64:
Kindly add references after this statement: “Previous studies in LMIC have reported links between mental health disorders and multimorbidity.”

***Response:***

We have added references to this statement:

*Previous studies have reported links between mental health disorders and multimorbidity [20, 21].. (Page 3, line 66; and Page 5, line 67)*

Methods
1. Line 93:
Perhaps start the sentence with “The” instead of “Our”.

***Response:***

This has been revised as suggested and now it reads:

*…The long-term conditions study in rural and urban Malawi is an ongoing… (Page 5, line 98)*

2. Line 110:
Please define what “usual members” are.

***Response:***

We have defined usual/resident members as follows:

*Individuals aged 15 years and above, who were usual members/residents of households in the rural or urban HDS were eligible for recruitment. Usual members were defined as individuals who live in a household within the surveillance areas for a significant portion of the year and consider it their primary place of residence. We recruited adolescents to understand better the early risk factors for long-term conditions and their developmental origins, given that the previous survey [42] already identified measurable prevalence of obesity, hypertension and diabetes at age 18 years.(Page 5, line 112-114; Page 6, line 115-117)*

3. Line 116:
Kindly explain the significance of the thumbprint consent. Was this for participants who were unable to give written consent because they were unable to read or write?

***Response:***

We have added the explanation of why a thumbprint was used i.e., for those unable to write as follows:

*…written (or a thumbprint for individuals unable to write) consent from participants… (Page 6, line 124 - 125)*

4. Line 123:
Should be “(PHQ-9)” and not “(PHQ)-9”.

***Response:***

We have revised this.

5. Lines 127-128:
Are there categories associated with the said scores? If so, please describe them instead of saying “with higher scores corresponding to more symptoms and increasing severity of depression.”

***Response:***

We greatly value your insights regarding the description of the PHQ-9 in our manuscript. In our commitment to clarity and thoroughness, we have provided a concise yet comprehensive description of the PHQ-9, supported by a pertinent reference, to ensure the integrity of our approach. As explained, the use of scale scores as continuous variables, rather than dichotomising into, for example, depressed/not depressed, retains more information and better reflects the continuous distribution of symptoms of common mental disorder in community settings.

Simultaneously, we wish to bring to your attention that, during our analysis, we were actively engaged in validating our scales within the Malawian context. Although this validation work is nearing completion, its findings are part of a separate body of work and, as such, cannot be incorporated into the present paper.

However, it may be of interest to note that in these validation studies, for identification of DSM 5 current Generalised Anxiety Disorder, at the optimum cut off of ≥6, in the rural site the GAD7 had sensitivity of 62% and specificity of 76%; in the urban site sensitivity was 84% and specificity was 77%. For identification of DSM 5 current Major Depressive Disorder, at the optimum cut off of ≥8, in the rural site the PHQ9 had sensitivity of 70% and specificity of 71%; in the urban site sensitivity was 81% and specificity was 80%.

6. Lines 132-133:
Are there categories associated with the said values? If so, please describe them instead of saying “with higher values indicating more symptoms and more severe anxiety.”

***Response:***

Please see our response to item 5 above.

7. Line 137:
Kindly add reference after the multimorbidity definition.

***Response:***

Reference has been added.

8. Overall:
Despite ongoing enrolment and data collection at the study sites, the methods (what was done for purposes of this particular study) need to be written in the past tense. For example, Lines 110-111 “Individuals who are aged 15 years and above and who are usual members/residents of households (in the rural or urban HDSS) are eligible for recruitment.”

***Response:***

We have revised this section and other relevant sections to include past tense. The changes in the methodology section to reflect this are as follows:

*Data collection was interviewer-led using electronic data capture with Open Data Kit software [42]. Questions were read to each participant in the language of their choice. Most interviews in Karonga were conducted in Chitumbuka, those in Lilongwe were mostly in Chichewa. All field workers were trained using standard operating procedures, including obtaining informed, written (or a thumbprint for individuals unable to write) consent from participants, biomeasures and venepuncture. (Page 6, line 118 - 125)*

Results

1. Overall:
Results are explained in detail, appropriate statistical tests used, and thanks to the authors for conducting the sensitivity analyses!

**Response:**

Thank you.

Discussion

1. Line 288:
The words: “for urban” missing after “95% CI 9.4 – 19.1%.”

***Response:***

We have added “for urban”. Thanks for noting that.

2. Lines 295-296:
Kindly provide more information on these mentioned “previous findings” – where was the study conducted? Are the sociodemographic factors such as age, sex, etc similar to your study?

***Response:***

We have revised this section to add in more information about the studies cited and their sociodemographic comparisons. The section now reads as follows:

*In our study, we observed a strong positive correlation (ρ = 0.68) between scores on the PHQ-9 and GAD-7 scales. This finding is similar to previous research conducted in the United Kingdom and Bangladesh, which reported correlations of 0.65 and 0.75, respectively, between these two scales [61-63]. These results suggest PHQ-9 and GAD-7 may have broad application across different settings with inherent sociodemographic differences. The strong correlation between scores also reflects the common co-existence of these conditions [64, 65], overlapping symptoms [66] and shared underlying neurobiological and psychological mechanisms [67, 68]. (Page 16, line 308 - 314)*

3. Lines 303-304:
What was the systematic review on? Was it conducted on studies in LMICs as well?

***Response:***

The systematic review was on multimorbidity and depression and yes, it included studies from LMIC. We have added a few words to capture this:

*A systematic review on multimorbidity and depression that included studies from LMIC reported... (Page 17, line 319-320)*

4. Line 305:
Were these 42 countries LMICs too?

***Response:***

Yes, there were several countries from LMICs and high income too. We have edited to capture that the study included LMIC as well:

*Similarly, a study encompassing 42 countries (including LMIC) [71] showed that … (Page 17, line 322 - 323)*

5. Overall:
Perhaps provide comparisons with studies conducted in other LMICs other than India and South Africa only.

***Response:***

Thank you for your insightful comments. In our discussion section, we have made comparisons between our findings with India, South Africa, Bangladesh, and findings from several systematic reviews which covered studies from LMICs. Further to addressing this comment, we have also made comparisons to other studies from high income countries e.g., the UK. We hope that this has helped improve our discussion section:

*In our study, we observed a strong positive correlation (ρ = 0.68) between scores on the PHQ-9 and GAD-7 scales. This finding is similar to previous research conducted in the United Kingdom and Bangladesh, which reported correlations of 0.65 and 0.75, respectively, between these two scales [61-63]. These results suggest PHQ-9 and GAD-7 may have broad application across different settings with inherent sociodemographic differences. The strong correlation between scores also reflects the common co-existence of these conditions [64, 65], overlapping symptoms [66] and shared underlying neurobiological and psychological mechanisms [67, 68]. (Page 16, line 308 - 314). (Page 17, line 316-324)*

Overall:

Well done to the authors for writing such a relevant manuscript with appropriate statistical methods. It has been an informative and interesting read.

***Response:***

Thank you for the insightful comments that have improved our manuscript.

**Reviewer #2: Manuscript number: PGPH-D-23-02092**

Journal: Plos Global Public Health
Thank you for the opportunity to review the manuscript titled “Association between physical multimorbidity and common mental health disorders in rural and urban Malawian settings: preliminary findings from Healthy Lives Malawi long-term conditions survey” on behalf of PloS Global Public Health.
Multimorbidity is an area of increasing concern. As the authors state, there is very little information available about multimorbidity in low and middle income (LMIC) contexts, and even less so in low-income contexts.
I would like to commend the authors on a thorough, clearly described and well written paper. The authors make a good argument for why country-specific studies on multimorbidity are needed and the paper’s focus on multimorbidity in Malawi is welcome. The additional focus on mental health is also welcome as this is known to be an underserviced area in many LMICs.
The Methods are well described, and the authors make use of a rich dataset, which can be difficult to find in LMIC contexts; thereby relaying important results about this population.
The results are well laid out. The finding of 16% prevalence in young people in Malawi is quite interesting and perhaps unexpected.
The paper is important and provides baseline information from a large dataset in Malawai. I feel that it is also highly relevant to the journal and the collection of articles (multimorbidity in Africa).
I would advise in future that the authors consider conducting a latent class analysis or cluster analysis to interpret the disease groupings. I look forward to seeing more research come out of this study.

***Response:***

Thank you for reviewing our manuscript. Your comments and suggestions have helped improve our manuscript.

Minor
Abstract
- Line 23 is missing the word “disease” or “condition”.

***Response:***

We have added this as follows:

*Multimorbidity was defined as having two or more measured (diabetes, hypertension) or self-reported (diabetes, hypertension, disability, chronic pain, HIV, asthma, stroke, heart disease, and epilepsy) conditions. (Page 2, line 23)*

Methods
- Line 110: Although I know why there is a focus on individuals 15 years and older, in many of my own articles I have been asked by reviewers to explain why this age group starts at 15 years rather than 18 years. I think it would be good to explain the significance of starting the observations with people aged 15 years.

***Response:***

We have revised the section to capture why it is important to observe individuals aged 15 years and older and now the section reads as follows:

*Individuals aged 15 years and above, who were usual members/residents of households in the rural or urban HDS were eligible for recruitment. Usual members were defined as individuals who live in a household within the surveillance areas for a significant portion of the year and consider it their primary place of residence. We recruited adolescents to understand better the early risk factors for long-term conditions and their developmental origins, given that the previous survey [42] already identified measurable prevalence of obesity, hypertension and diabetes at age 18 years.(Page 5, line 112-114; Page 6, line 115-117)*

- Line 120: Was there a reason that data collection between the two sites did not occur at the same time instead of a year apart?

***Response:***

The Karonga site, with its well-established Health and Demographic Surveillance System (HDSS) operations since 2002, presented a convenient starting point for our data collection. The existing enumeration of the population facilitated the initiation of the study. This site also provided valuable insights for refining field procedures and the overall administration of the study. Conversely, in Lilongwe, we had to establish an urban HDSS from the ground up before commencing the long-term conditions survey, which necessitated a more extended period for system establishment and stabilization in the urban area, a process that was considerably delayed by COVID.

- Line 134: Have the tools been validated in Malawi before? If not, I think your validation of the tool deserves to be highlighted more.

***Response:***

Yes, the tools have been validated and the validation exercise has been completed and the work will be published as a separate piece of work. Our validation results of the two scales have indicated the following: For identification of DSM 5 current Generalised Anxiety Disorder, at the optimum cut off of ≥6, in the rural site the GAD7 had sensitivity of 62% and specificity of 76%; in the urban site sensitivity was 84% and specificity was 77%. For identification of DSM 5 current Major Depressive Disorder, at the optimum cut off of ≥8, in the rural site the PHQ9 had sensitivity of 70% and specificity of 71%; in the urban site sensitivity was 81% and specificity was 80%.

- Line 147 – 150: Please reference the standards that were used to determine the cut-offs for diabetes and hypertension.

***Response:***

We have provided references for the standards used in determining cut-offs for diabetes and hypertension.

- Line 195: Please fix the sentence, it should be for people aged 15 to 17 years of age. “For participants aged between under 18 years, written informed consent from their legally acceptable representative”.

***Response:***

The section has been revised.

Results
- I appreciate that Table 1 is quite a busy table. However, could the places where p-values were significant be highlighted in some way. I note that it is stated in a footnote however I would like to be able to look at the table and be able to see it immediately.

***Response:***

Thank you for your valuable suggestion regarding the inclusion of p-values in our table. We appreciate your attention to detail and agree that indicating p-values could enhance the comprehensiveness of our data presentation. However, as you have also observed, the table is currently quite detailed with stratification by site and sex, creating a complexity that might be compounded by the addition of p-values. We have carefully considered the balance between detail and readability and concluded that the footnotes adequately convey the necessary statistical information. Additionally, as the primary purpose of this table is to present descriptive data, we believe its current format effectively meets this objective. Thank you again for your thoughtful feedback.

Discussion

- Are the authors able to identify what diseases are driving the high prevalence of multimorbidity in young people in Malawi?

***Response:***

We have commented on the contributors of high prevalence of multimorbidity in Malawi and revised the discussion section as follows:

*These preliminary data, from a large, ongoing cross-sectional study in urban and rural Malawi, show a link between multimorbidity from physical health conditions and increased depression and anxiety symptoms. Notably, the greater the number of co-morbid physical health conditions, the greater the depression and anxiety symptom scores. We observed multimorbidity in 16% of young adults (< 30 years ) in rural and urban areas, which is comparable to the 13% reported in South Africa within the same age group [56]. The most prevalent long-term conditions in young adults were asthma and chronic pain, followed by disability, hypertension, HIV and epilepsy, with hypertension and disability the most common combination. With the increasing burden of cardiometabolic conditions as the population ages, our findings suggest that, in the future, a high proportion of the Malawian adult population will be living with multiple long-term health conditions.* (Page 16, line 288-300)
